# Dalbavancin for treatment of implant-related methicillin-resistant *Staphylococcus aureus* osteomyelitis in an experimental rat model

**DOI:** 10.1038/s41598-018-28006-8

**Published:** 2018-06-25

**Authors:** Manuel Kussmann, Markus Obermueller, Florian Berndl, Veronika Reischer, Luzia Veletzky, Heinz Burgmann, Wolfgang Poeppl

**Affiliations:** 1 Department of Internal Medicine I, Division of Infectious Diseases and Tropical Medicine, Medical University Vienna, Vienna, Austria; 2 Military Medical Cluster East, Austrian Armed Forces, Vienna, Austria

## Abstract

Dalbavancin is a new semisynthetic lipoglycopeptide with improved antimicrobial activity against various gram-positive pathogens. It demonstrates an extensive plasma half-life which permits outpatient parenteral antimicrobial therapy with weekly intervals and might therefore be an excellent treatment alternative for patients requiring prolonged antimicrobial therapy. The present study investigated dalbavancin monotherapy in an experimental implant-related methicillin-resistant *Staphylococcus aureus* (MRSA) osteomyelitis model. A clinical MRSA isolate and a Kirschner-wire were inserted into the proximal tibia of anaesthetized Sprague-Dawley rats. Four weeks after infection 34 animals were treated over 4 weeks with either dalbavancin (20 mg/kg loading-dose; 10 mg/kg daily), vancomycin (50 mg/kg twice daily) or left untreated. Twenty-four hours after the last treatment dose tibial bones and Kirschner-wires were harvested for microbiological examination. Based on quantitative bacterial cultures of osseous tissue, dalbavancin was as effective as vancomycin and both were superior to no treatment. No emergence of an induced glycopeptide-/lipoglycopeptide- resistance was observed after a treatment period of four weeks with either dalbavancin or vancomycin. In conclusion, monotherapy with dalbavancin was shown to be as effective as vancomycin for treatment of experimental implant-related MRSA osteomyelitis in rats, but both antimicrobials demonstrated only limited efficacy. Further studies are warranted to evaluate the clinical efficacy of dalbavancin for the treatment of periprosthetic *S. aureus* infections.

## Introduction

The increasing prevalence of drug-resistant gram-positive cocci, such as methicillin-resistant *Staphylococcus aureus* (MRSA) has complicated the management of difficult to treat infections^1–3^. Dalbavancin, a new semisynthetic lipoglycopeptide, is currently approved for single-dose treatment of acute bacterial skin and soft tissue infections in adults. Dalbavancin possesses unique pharmacokinetic properties with an extensive plasma protein binding and a prolonged half-life (t_1/2_) of 181–216 hours^4–6^. The *in vitro* activity of dalbavancin against various gram positive pathogens including MRSA and vancomycin-intermediate *S. aureus* (VISA) isolates was shown to be even improved when compared to established antibiotics such as linezolid, teicoplanin or vancomycin^7^.

For implant-related MRSA-infections, in particular in osseous tissue, current treatment strategies recommend a combination of a biofilm active substance like rifampicin or fosfomycin together with vancomycin^8,9^. In a recent study it was shown that dalbavancin was equally active in an MRSA-osteomyelitis model compared to vancomycin^10^. However, hitherto no data on the efficacy of dalbavancin in the treatment of implant-related osteomyelitis is available. Thus, the present study set out to investigate the efficacy of dalbavancin compared to vancomycin in an experimental implant-related MRSA osteomyelitis rat-model.

## Results

During induction of the experimental implant-related MRSA osteomyelitis, two out of 36 rats died during anesthesia and were not further analyzed. Thus, 12 animals remained in the dalbavancin group and 11 animals in the vancomycin and control group, respectively.

**Figure 1:**
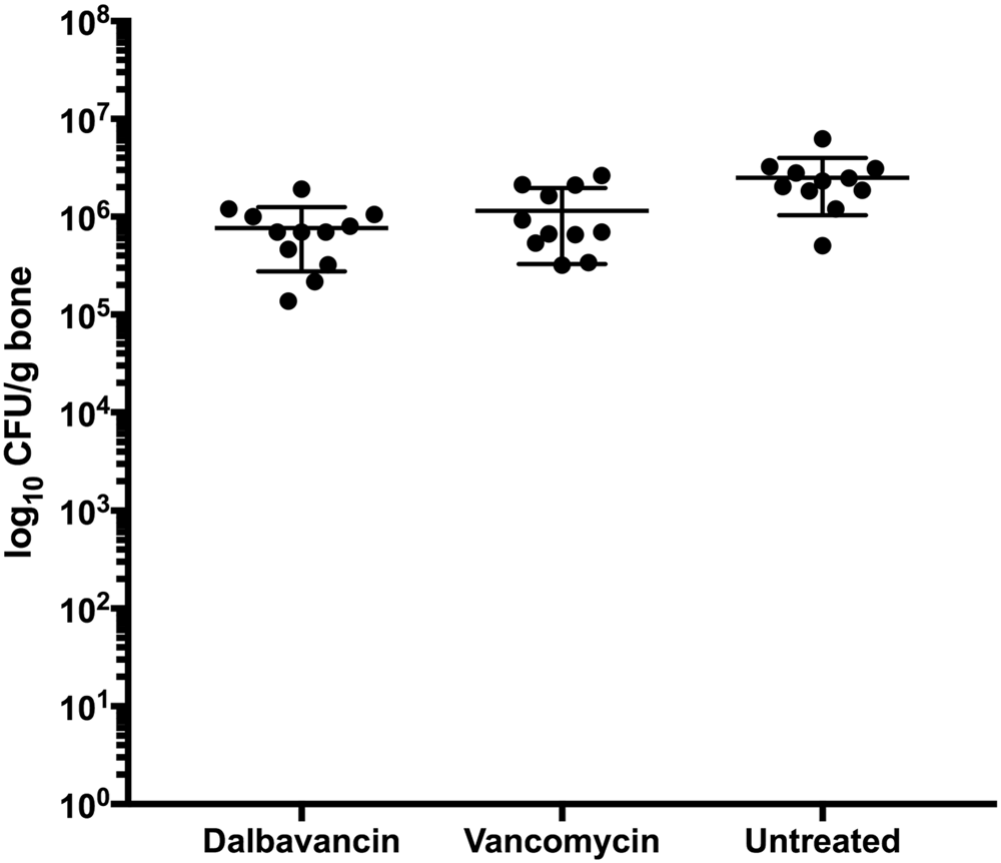
Mean bacterial counts in log_10_ CFU/g bone (±SD) from osseous tissue after a four-week treatment period with dalbavancin, vancomycin or without treatment in an experimental implant-related MRSA osteomyelitis in rats. The three groups, dalbavancin, vancomycin and untreated, comprised twelve, eleven and eleven rats, respectively.

All bacterial cultures from bone and Kirschner-wires were found positive for MRSA. The mean (±SD) bacterial counts in osseous tissue were 5.96 log_10_ CFU/g bone (±0.32 log_10_ CFU/g bone) for vancomycin, 5.79 log_10_ CFU/g bone (±0.33 log_10_ CFU/g bone) for dalbavancin and 6.33 log_10_ CFU/g bone (±0.28 log_10_ CFU/g bone) for untreated animals (Fig. 1). Significant differences were shown for dalbavancin (p < 0.01) and vancomycin (p = 0.03) when compared to no treatment but not between the two antimicrobial therapies. The mean (±SD) body weight increase was 36.8 g (±8.6 g) in the dalbavancin group, 4.3 g (±9.4 g) in the vancomycin group and 47.4 g (±33.4 g) in the untreated group. Thus, the body weight increase in the dalbavancin (p < 0.001) and the untreated control group (p < 0.001) were significantly higher when compared to the vancomycin group. All isolates obtained from antibiotic treated bone samples and Kirschner-wires demonstrated MICs of 0.064 mg/L for dalbavancin and 1.5–2 mg/L for vancomycin. Thus, no emergence of induced glycopeptide/lipoglycopeptide resistance occurred in the present study.

## Discussion

The similar efficacy of dalbavancin and vancomycin observed in the present study is in line with a previous study by Barnea *et al*.^10^. Using an experimental sternal MRSA osteomyelitis rat model, they demonstrated a decrease of >3 log_10_ CFU/g bone after a treatment period of two weeks with either dalbavancin or vancomycin compared to untreated animals^10^. Thus, in terms of bacterial loads in osseous tissues, the efficacy of the antimicrobial treatment was more distinct in that osteomyelitis model without foreign body^10^. Several factors might contribute to this discrepancy. The MICs of the bacteria used, however, were identically for dalbavancin (0.06 mg/L) and differed only minimally for vancomycin (1.5 mg/L vs 1.0 mg/L)^10^. Also, with exception of the treatment period of two versus four weeks, the dosing regimens for dalbavancin and vancomycin were identically^10^.

Several *in vitro* and *in vivo* studies demonstrated that the antibiotic concentrations needed for biofilm eradication were approximately 10 to 1000 times higher than the MICs or even minimal bactericidal concentrations (MBCs) of planktonic cells^3,11–13^. Especially in low penetration compartments such as osseous tissue, these concentrations are often impossible to reach which results only in biofilm suppression but not in eradication^3,13^. Thus, formation of mature biofilm on the implanted Kirschner-wires might be the most suitable explanation for the low efficacy of dalbavancin and vancomycin monotherapy observed in the present study. With regard to previously performed experiments, median bacterial counts of implanted wires were only 2.5 × 10^3^ CFU/implant (4.0 × 10^2^ to 4.5 × 10^4^) and 7.50 × 10^2^ CFU/implant (0 to 7.00 × 10^3^) in the untreated control groups^14,15^. Therefore, it is unlikely that only a contamination through the implanted wires was responsible for the high bacterial counts obtained from the osseous tissue in the present study.

In humans, 500 mg to 1000 mg dalbavancin once weekly are usually applied to patients requiring prolonged antimicrobial therapy. Among non-infected orthopedic patients receiving a single dose of 1000 mg dalbavancin, the bone concentrations obtained were 6.3 µg/g (±3.1 µg/g) after 12 hours, 3.8 µg/g (±2.7 µg/g) after 7 days and 4.1 µg/g (±1.6 µg/g) after 14 days^16^. These concentrations indicate sufficient drug levels in osseous tissue for treatment of dalbavancin susceptible organisms^16^. The rationale for the treatment regimens used in the present study was to achieve bone levels of drug closely mimicking those obtained in humans. In rats, for dalbavancin a loading dose of 20 mg/kg followed by 10 mg/kg o.d. were shown to result in comparable bone levels to a single i.v. dose of 1000 mg dalbavancin in humans^10,16^. However, it should be emphasized that bone concentrations of dalbavancin were assumed to have reached similar levels to those observed by Barnea *et al*. and were not determined in the present study^10^.

Of interest, no emergence of dalbavancin resistant *S. aureus* isolates was observed after a treatment period of four weeks. This supports previous observations that development of dalbavancin resistance is rare^17^. According to current recommendations, first-line treatment of orthopaedic implant-associated infections caused by MRSA still includes glycopeptide antibiotics such as vancomycin or teicoplanin^8,18^. Thus, in regard to the similar activity to vancomycin, observed in the present study, and the unique pharmacokinetic properties, excellent for an outpatient parenteral antimicrobial therapy, dalbavancin might be a feasible alternative for long-term antimicrobial therapy of implant-related osteomyelitis.

## Conclusion

In summary, monotherapy with dalbavancin was as effective as vancomycin for treatment of experimental implant-related MRSA osteomyelitis in rats but both antimicrobials demonstrated only limited efficacy. Further studies are warranted to investigate the clinical efficacy of dalbavancin for treatment of periprosthetic *S. aureus* infections, preferable in combination with biofilm active drugs.

## Methods

### Animals

Male Sprague-Dawley rats were housed in a temperature and light controlled facility in the level 2 biohazard area of the Institute of Biomedical Research, Medical University of Vienna, Austria. All procedures were conducted according to guidelines for animal care and use of laboratory animals and the study protocol, including care and handling of the animals, was approved by institutional Animal Welfare Committee (Medical University of Vienna, Austria).

### Pathogen

The bacterial strain used was a clinical MRSA isolate (40496/08) obtained by routine microbiological examination from a patient with chronic osteomyelitis. Minimal inhibitory concentrations (MICs) for vancomycin (1.500 mg/L) and dalbavancin (0.064 mg/L) were determined by agar disk diffusion method with Etests (Liofilchem SRL, Italy) on Mueller Hinton agar (MHA) plates and were assessed after an incubation of 18–24 hours at 36 °C (±1°C). For dalbavancin, additionally, a recently published broth microdilution (BMD) assay, in accordance to CLSI and EUCAST convenients, was used for validation of the obtained MICs (0.063 mg/L)^19^.

### Experimental animal model

Implant-related MRSA osteomyelitis was established in 36 Sprague-Dawley rats (Charles River WIGA GmbH, Sulzfeld, Germany) weighing 448 ± 29 g as described previously^14,15^. Briefly, bacteria were grown overnight in trypticase soy broth (TSB), diluted 1:100 in fresh TSB and incubated for five hours at 36 °C (±1 °C) to achieve exponential growth phase. An inoculum of 10 µl (1–5 × 10^6^ CFU/bone) of the bacterial suspension and a Kirschner-wire of 1 cm length were inserted into the left proximal tibia of each animal. Bacterial counts were validated before and after surgical procedures using viable counts.

Four weeks after induction of infection animals with radiographically confirmed osteomyelitis of the tibia were randomly assigned to receive either i.p. dalbavancin (Xydalba; Angelini Pharma, Vienna, Austria) 20 mg/kg loading dose followed by 10 mg/kg daily, i.p. vancomycin (Vancomycin Hikma; Hikma Farmaceutica, Terrugem, Portugal) 50 mg/kg twice daily, or no treatment over a period of 4 weeks.

According to manufacturer’s instructions, dalbavancin and vancomycin were reconstituted using aqua ad iniectabilia and further diluted with 5% dextrose solution or aqua ad iniectabilia, respectively.

The dosage regimen was based on drug concentrations in osseous tissue obtained in previous studies in rats closely mimicking bone concentrations in humans^10^.

### Bacterial cultures

Twenty-four hours after the last treatment dose, rats were euthanized and tibial bones were harvested and homogenized using a liquid-nitrogen-cooled CryoMill (6770 Freezer Mill; Spex SamplePrep, Metuchen, NJ). Quantitative cultures on Columbia sheep blood (COS) agar plates were performed for bone suspensions. Before homogenizing of the tibial bones, Kirschner-wires were removed, sonicated in sterile saline for qualitative cultures and rolled on COS agar plates. Recovered bacteria of all antibiotic treated animals (n = 23) were tested for the emergence of antimicrobial resistance by agar disk diffusion method using vancomycin and dalbavancin Etests.

### Statistical analysis

Results were analyzed using GraphPad Prism version 6. For comparison of the three treatment groups in regard to the mean bacterial counts obtained from osseous tissue and the body weight increase during the study period, the Kruskal-Wallis test with a post hoc analysis by Dunn’s multiple comparison test was used.

### Ethical approval

The study protocol, including care and handling of the animals, was approved by institutional Animal Welfare Committee of the Medical University of Vienna, Austria (ID 66. 009/0242-MF/V/3b/2016).
